# Towards the Standardization of Intestinal In Vitro Advanced Barrier Model for Nanoparticles Uptake and Crossing: The SiO_2_ Case Study

**DOI:** 10.3390/cells11213357

**Published:** 2022-10-25

**Authors:** Olimpia Vincentini, Valentina Prota, Serena Cecchetti, Lucia Bertuccini, Antonella Tinari, Francesca Iosi, Isabella De Angelis

**Affiliations:** 1 Department of Food Safety, Nutrition and Veterinary Public Health, Istituto Superiore di Sanità, 00161 Rome, Italy; 2 Department of Environment and Health, Istituto Superiore di Sanità, 00161 Rome, Italy; 3 Core Facilities, Microscopy Area, Istituto Superiore di Sanità, 00161 Rome, Italy; 4 Center for Gender Medicine, Istituto Superiore di Sanità, 00161 Rome, Italy

**Keywords:** intestinal absorption, in vitro intestinal triculture model, mucus layer, microfold cells intestinal barrier permeability, model standardization, nanoparticles

## Abstract

Increasing interest is being addressed to the development of a reliable, reproducible and relevant in vitro model of intestinal barrier, mainly for engineered nanomaterials hazard and risk assessment, in order to meet regulatory and scientific demands. Starting from the consolidated Caco-2 cell model, widely used for determining translocation of drugs and chemicals, the establishment of an advanced intestinal barrier model with different level of complexity is important for overcoming Caco-2 monoculture limitations. For this purpose, a tri-culture model, consisting of two human intestinal epithelial cells (Caco-2 and HT29-MTX) and a human lymphocyte B cell (Raji B), was developed by several research groups to mimic the in vivo intestinal epithelium, furnishing appropriate tools for nanotoxicological studies. However, tri-culture model shows high levels of variability in ENM uptake/translocation studies. With the aim of implementing the standardization and optimization of this tri-culture for ENM translocation studies, the present paper intends to identify and discuss such relevant parameters involved in model establishment as: tri-culture condition set-up, barrier integrity evaluation, mucus characterization, M-cell induction. SiO_2_ fluorescent nanoparticles were used to compare the different models. Although a low level of SiO_2_ translocation is reported for all the different culture conditions. a relevant role of mucus and M-cells in NPs uptake/translocation has been highlighted.

## 1. Introduction

Engineered nanomaterials (ENMs of at least one dimension ≤ 100 nm) are increasingly present in drugs and consumer products due to the innovative commercial and technical opportunities that they can offer. Conversely, concerns about ENMs’ impact on human health and the environment have been raised by scientists and regulators, who have stressed the need to properly assess and manage any potential risk linked to the nano-specific properties. 

It is commonly considered that traditional assays for chemical safety assessment are also suitable for ENM risk evaluation, albeit with necessary adaptations [1]. Fadeel and co-authors, suggest that simple adaptation could be insufficient and a speedup of EMN testing is needed preferentially using advanced in vitro tools [2]. The development of advanced in vitro models is a fast-moving area in which human relevant systems, more representative of the in vivo situation, are considered for hazard and risk assessment, followed by exposure to chemical substances including ENMs [3]. In the meanwhile, an in vitro approach fulfils European and International commitment towards the reduction of animal use in scientific research in line with the 3R principle, coupled with the shift from a phenomenological to a mechanistic approach [4,5]. From a regulatory perspective, it is important to verify if these models are robust enough to fulfil the regulatory requirements of inter-laboratory reproducibility and protocol transferability and, mainly, predictability of in vivo and/or human exposure data for specific endpoints.

Among the different routes of exposure to ENMs, oral ingestion is one of the most relevant and it is considered crucial for the investigation of the biological effects of ENMs. Ingestion of food containing ENM is the primary source of exposure since they are widely used as food additives to enhance food organoleptic properties or improve quality and safety [6,7,8]. ENMs are also used in food packaging to improve product stability and biodegradability of packages. The gastrointestinal tract (GIT) is considered the primary target organ for EMNs once ingested. Moreover, a small fraction of inhaled nanoparticles (NPs) can move to the GIT by systemic circulation through both muco-ciliary clearance mechanisms and alveolar barrier crossing, causing indirect toxic effects at the intestinal level [9]. 

After ingestion along the GIT, ENMs are exposed to the different physiological conditions existing in mouth, oesophagus, stomach and intestine [10,11]. Exposure to these conditions changes the ENM properties (size, aggregation/agglomeration, or alteration of the dispersion stability) which in turn can influence NPs’ behaviour at the intestinal barrier level as well as their potential toxicity. Recently, European and international regulatory agencies, such as EFSA or ISO, have emphasized the importance of hazard identification to investigate ENMs’ dissolution profile in the digestive tract and their (eventual) translocation through the intestinal barrier [12,13]. In this respect, some artificial digestion models were developed using artificial matrixes simulating the different human digestion fluids. These have been successfully applied to different types of ENMs either with dynamic or static conditions of incubation [14,15,16,17].

In vitro models of the intestinal barrier are long-established and transversally applied in many research fields. In particular, Caco-2 cells grown on inserts are a well-established model of human enterocytes, widely used for determining active and passive absorption of drugs and chemicals [18]. However, the culture has some limitations especially because it does not properly mirror the structural complexity of the in vivo gut environment. Caco-2 cells are of colonic origin and display junctions tighter than those present in the small intestine, thus causing less permeability through the paracellular route, but they also exert an overexpression of P-Glycoprotein which limits absorption to compounds transported by the carrier [19,20]. The use of co-cultures with different levels of complexity, which combine different types of intestinal cell lines, has greatly expanded the application capabilities of the model [21,22]. For instance, the simultaneous presence of mucus-secretory cells (HT29-MTX), due to the relevance of mucus defensive properties and impacts on nanoparticle mobility, and hematopoietic cells (Raji-B cells), able to promote Caco-2 conversion in specialized microfold cells (M-cells) involved in particulate uptake, can enhance the physiological relevance of the model [23,24]. 

M-cells are located in the follicle-associated epithelium of Peyer’s patches and highly specialized in the phagocytosis and transcytosis of gut lumen macromolecules, particulate antigens and pathogenic or commensal microorganisms across the epithelium [25]. Functionally, they transport particulate matter from the gut lumen across the epithelial barrier to allow sampling by antigen-presenting cells of the immune system, which traffic through the extracellular lymph fluid on the basolateral side [26]. Overall, M-cells represent approximately 1% of the cells lining the intestine and their phenotype has largely remained uncharacterized, due to difficulties in identifying and isolating sufficient quantities of this sparsely occurring cell population [27].

M-cell morphology is characterized by the lack of apical microvilli in the intestine and absence of cilia in the upper airway, a feature that could be helpful for luminal microparticles in binding apical capture receptor, but this is not the sole responsible factor. In fact, epithelial microvilli house a multitude of glycoproteins and associated carbohydrates on the apical membrane. Little is known about the proteins expressed on the apical surface of M-cells, which show a different degree of glycosylation than enterocytes [28]. The reduction of the carbohydrate component can reduce the electrostatic repulsion, allowing microparticles to get closer to the M-cell apical membrane [29]. On their basolateral surface, M-cells possess a membrane invagination, which acts as a “pocket”, forming a specialized microenvironment containing B- and T-lymphocytes, macrophages and dendritic cells. This component provides a docking site for lymphocytes and other antigen-presenting cells, reducing the distance between the apical and basolateral surfaces from which trans-cytotic vesicle transport begins, also playing a role in intracellular communication [30]. 

Presence of B cells seems to be an essential requirement for development of peculiar M-cell functions, in particular the establishment of a polarized cellular machinery able to capture a large cargo at the apical membrane and transport it to the basolateral end for delivery to dendritic cells. For this purpose, they reorganized tight junction (TJ) structures to allow large endocytosis. This activity is specifically directed at particulate matters in the range of micro/nano dimension (for example, bacteria and viruses) [31].

The possibility of obtaining “M-like’ cells through in vitro enterocytic conversion after lymphocyte stimulation provides a great opportunity to recreate a more physiological model of intestinal mucosa, since the in vivo M-cell population is scarcely characterized, due to their limited presence [25]. However, identification of specific markers for characterization and identification of these cells is still challenging. In fact, in the in vitro model represented by the Caco-2 /Raji B co-culture, most of the genes selectively expressed by natural intestinal M-cells are not induced [30] Recently, in murine enterocyte cultures the cytokine receptor activator of NFK-ligand (RANKL) and the transcription factor Spi-B have been shown to be required for the differentiation of M-cells from epithelia stem cells [32]. 

Globet cells are considered the second most prevalent phenotype, after enterocytes, in the intestinal mucosa. They are responsible for the production of mucus, which covers the epithelium and regulates the exchange of water, gases or nutrients, as well as having a protective function for the gastrointestinal tract by preventing (nano)particles or pathogen penetration [33]. 

Mucins are the main structural components of mucus. They are high molecular weight glycoproteins linked to each other by disulfide bonds forming a network able to sieve varied sizes of (nano)materials that come in contact with the mucus layer. Mucins are categorized into distinct families (Muc 1, Muc 2, Muc 3, etc.) and roughly classified into acid or neutral mucins, based on their carbohydrate chemical composition. The neutral mucins can be found primarily in the surface epithelia of the stomach and Brunner’s glands of the duodenum. The acid mucins are found widely distributed throughout the gastrointestinal tract [34]. Important levels of MUC5C mRNA are found in the HT29-MTX cell line, with the level increasing dramatically between 7 and 14 days of culture. Conversely, a similar increase in expression was not detected in the Caco-2 cell line where the expression remains low [35].

Some ENM properties can drive the interaction with mucus, such as size, chemical composition, surface charge and ligand density; ENMs with size below 50 nm easily penetrate the mucus layer and several biological effects on the intestinal mucus have been reported for a panel of different ENMs [36].

Consequently, the absence of mucosal component in the in vitro model does not allow an accurate and reliable assessment of ENMs’ absorption profile [34,37].

In order to construct an in vitro model that better respects the composition and characteristics of the intestinal mucosa in vivo, a co-culture model was established consisting of Caco-2 and HT29-MTX cells, such as mucus-secreting cells [38]. Mature HT29-MTX cells are obtained by methotrexate treatment of the HT29 adenocarcinoma cell line [39]. According to previous works, the 9:1 ratio (Caco-2:HT29-MTX) was selected [20,40]. The presence of Raji B cells, derived from a human lymphoma, induce the transformation of Caco-2 cells in M-cells [40], so a tri-culture model has been proposed by several research groups to better mirror the in vivo intestinal epithelium and furnish suitable tools for nanotoxicological studies [21,22,27,41].

The co-culture models developed so far show high results variability in ENM uptake/translocation studies. This variability is related to the different conditions of preparation and culture maintenance used in different laboratories. It is therefore extremely advisable to promote standardization and optimization of a Caco-2 absorption model for ENM translocation studies [42,43].

The resent paper intends to identify and critically discuss the relevant parameters involved in the establishment of the intestinal barrier tri-culture model with the aim of promoting the protocol robustness for improvement of its inter-laboratory transferability. Particularly, protocol improvements have been focused on: (i) identification of the best procedure for tri-culture model set-up and M-cell phenotype induction(ii) definition of some benchmarks (barrier integrity, mucus production, M-cell identification) to evaluate model performances. Moreover, some important aspects and limits of the proposed model are also evaluated, as for instance the influence of the pore dimension of the trans-well membrane in ENM translocation. In this respect, the trans-well system on its own may represent a limiting factor for ENM passage, depending on several characteristics such as pore size and density and, to a lesser degree, by their direct interaction with the tested materials [44]. In general, 3 µm pore size inserts are the more commonly used filters for ENM translocation studies, since this pore dimension ensures the passage of particles even in aggregate form [22,45], although Garcia Rodrigues and colleagues reported cell migration phenomena from the apical (Ap) to basolateral (Bl) chamber resulting in the formation of a double cell layer [41]. For this reason, it is noteworthy to make a careful evaluation of the interactions between NPs and empty inserts before proceeding with cellular barrier experiments [46,47]. 

Finally, SiO_2_ fluorescent NPs have been used to test the system, comparing their translocation through the Caco-2 monolayer and in the tri-culture model. These NPs were selected due to their large application in different market products including food, as food additive, and nanomedicine, as drug carriers. Moreover, they have been extensively characterized on the Caco-2 model in our previous studies in the frame of H2020 EU project NANoREG.

## 2. Materials and Methods

### 2.1. Cell Culture

Caco-2 cells (HT-B 37 clone) (human colorectal adenocarcinoma) and Raji B cells (human B lymphocyte) were obtained from the European Collection of Cell Culture (ECACC, UK); HT29-MTX E12 cells (human colorectal adenocarcinoma) were obtained from the American Type Culture Collection (ATCC, Manassas, VA, USA). 

Caco-2 (passage 5–12) and HT29-MTX (passage 38–45) cells were cultured in DMEM high glucose (Dulbecco’s modified Eagle medium) supplemented with 10% heat-inactivated Foetal Bovine Serum (FBS), 1% non-essential amino acids, 1% L-glutamine and 1% penicillin and streptomycin (PEST). Caco-2 and HT29-MTX-E12 were sub-cultured at 90% confluence once a week by dissociating with trypsin (Tryple L-select,) and seeded at a density 30 × 10^3^ /cm^2^ (split 1:6) and 25 × 10^3^/cm^2^ (split 1:10) respectively.

Raji B cells (passage 7–14) were cultured in suspension in RPMI high glucose supplemented with 10% heat-inactivated FBS, and 1% Penicillin/Streptomycin. Raji B cells were maintained at a cell density of 1 million cells/cm^3^ and sub-cultured only two times before performing the experiments. The cell lines were grown under standard incubation condition (37 °C 5% CO_2_). All the reagents used for the cultures were purchased from Gibco (Gibco-Thermo Fisher Scientific, Waltham, MA, USA).

### 2.2. Bi-coltures and Tri-Culture Model Assembly on Inserts

A monoculture of Caco-2 cells, a bi-culture of Caco-2/HT29-MTX cells and a bi-culture of Caco-2/Raji B cells were run in parallel to compare the performance of the Caco-2/HT29-MTX/ Raj B triculture model.

- Caco-2 monoculture on inserts was obtained by seeding 2.25 × 10^3^ cells on the Ap compartment of Polyethylene Terephthalate Transwells^®^ insert (PET) with a pore size of 1 and 3 μm (Millipore^®^) (Merck KGaA, Darmstadt, Germany), allocated in 12-well culture plates (Falcon) with 0.5 mL of complete culture medium in the Ap compartment, while Bl compartment was filled with 1.5 mL. DMEM complete culture medium was changed every two days.
- Caco-2/HT29-MTX bi-cultures were obtained by mixing and seeding the two cell lines at 9:1 ratio at a density of 2.25 × 10^5^ cells/cm^2^, in the Ap compartment. Inserts were then maintained as reported for Caco-2 monoculture.
- Caco-/Raji and Caco-HT29-MTX/Raji B co-cultures were obtained by adding at the 14th day of co-culture, 5 × 10^5^ Raji B cells in the Bl side of each insert in DMEM:RPMI (1:1) culture medium to allow M phenotype induction in Caco-2 cells. Raji B cells were completely removed and reseeded on day 16th and 19th of co-culture.

Moreover, in the present study the following three different conditions have been used to induce M-cell conversion, according to literature evidence:

1. Raji B cells seeded in the Bl compartment. Raji B cells were seeded in the Bl compartment and refreshed at 14th, 16th, and 19th days of culture to prevent their overgrowth which might cause degeneration of the lymphocyte’s population and, consequently, presence of necrotic factors in the model.
2. Simplified inverted culture method. In this case Caco-2 and Caco-2 /HT29-MTX cells were seeded on the Bl side of the insert and left for 48 h to allow cells’ adherence to the insert membrane before turning them upside down. Inverted cells were cultured for 14 days and then stimulated with Raji B cells placed in the Ap compartment. The inverted model required an elevated level of expertise coupled with a high risk of contamination during the turning phase of the inserts. For this reason, it was considered not suitable for the next steps in procedure standardization and will not be further discussed.
3. Raji B conditioned medium. To investigate if the direct contact between intestinal epithelial cells and lymphocytes is required to induce M-phenotype in Caco-2 cells, conditioned medium from Raji B cells was also used at each cycle of induction (14, 16 and 19 days of co-culture). Lymphocytes conditioned medium from Raj B cells was obtained as follows: the day before each induction, 330 × 10^3^ Raji B cells/mL Raji B cells were cultured in DMEM/RPMI (1:1) for 24 h, then the cellular suspension was centrifuged and 1.5 mL of the supernatant was added to the Bl compartment of the insert. This experiment has been investigated only with 3 µm pore size inserts to maximize the effect of diffusion factor(s).

Finally, to determine how long the M-phenotype was maintained in our experimental conditions after the last cycle of induction (19th day of culture), co-cultures were checked for the next 5 days for barrier integrity. We observed that the model was stable until the 22nd day of culture (data not shown).

### 2.3. Barrier Integrity 

Trans-epithelial Electrical Resistance (TEER) was evaluated by a chop-stick electrode device (Millicell ERSVoltameter-Millipore-Sigma, San Luis, Mo, USA). On day 20 of co-cultures, inserts were moved to a new 12-well culture plate, in fresh DMEM in both compartments. TEER measurements were performed at 37 °C. A cells free-insert (blank) was included. Three separate measures were performed for each insert. Results were expressed as ohms × cm^2^ according to the following formula (1):

TEER = [Ω cell monolayer − Ω filter (cell-free)] × filter area (1.12 cm^2^) (1)

### 2.4. Paracellular Permeability 

Paracellular permeability was assessed by fluorescein isothiocyanate dextran (FITC-dextran) 40 kDa (Sigma-Aldrich, Saint Louis, MO, USA) and by Lucifer Yellow CH di-lithium salt (LY) 457 D (Sigma-Aldrich) translocation. Briefly, after the 20th day of co-culture, the medium was removed from the inserts and Ap and Bl chambers were washed twice with Hanks Balanced salt solution Buffer (HBSS). Then 1mg/mL of FITC-dextran or 0.4 mg/mL of LY in 0.5 mL of HBSS were added in the Ap compartment while the Bl side was filled with 1 mL of HBSS. After 2 h incubation at 37 °C, 100 µL of the Bl volume from each insert were collected and transferred into a black microtiter 96 well-plate (Perkin Elmer, Waltham, MA, US). Samples were analyzed by spectro-fluorimetry (Nivo, Perkin Elmer, Waltham, MA, US) at λ 495 em λ 521 exc) nm for FITC dextran and λ 485–528 for LY. Results were expressed as Papp according to the following formula (2):

Papp = ((DQ/Dt) × V) × (1/AC0)(2)

where DQ/Dt is the amount of LY and FITC dextran transported in the Bl compartment per time unit (t), V is the Bl volume (cm³), A is the surface area of the filter (1.12 cm^2^) and C0 is the initial concentration in the Ap compartment.

### 2.5. Localization of Tight Junction ZO-1 Protein

Tight junction protein ZO-1 expression was assessed by laser scanning confocal microscopy performed on a Zeiss LSM 980 with Airyscan2, using the 40 × and 63 × oil objectives and excitation spectral laser lines at 405, 488, 546, 594 and 633 nm. Briefly, at the end of the differentiation process, trans-well membranes were washed twice with PBS and fixed for 10 min at cold temperature (+4 °C) with methanol. Following further washing, the samples were blocked with 2% *w*/*v* bovine serum albumin in PBS for 60 min at 37 °C, to prevent non-specific binding by antibodies. Mouse monoclonal anti-ZO-1 antibody (1:50, BD Biosciences Billerica, MA, USA) was then incubated for 1 h at 37 °C, followed by the appropriate Alexa fluor-conjugated secondary antibody (Thermo Fisher Scientific, Waltham, MA, USA). After further washing, the samples were counterstained with DAPI (Thermo Fisher Scientific, Waltham, MA, USA) for 10 min and then washed three times with PBS. Finally, the membranes were removed with a scalpel and placed on glass slides, before mounting coverslips with Vectashield (Vector Laboratories, Burlingame, CA, USA) mounting medium. Two independent experiments were performed for each condition. 

### 2.6. Mucus Characterization

#### 2.6.1. Mucus Staining

For acid mucins identification, on the 21st day of culture, culture medium was removed and 0.5 mL of Alcian blue staining (pH 2.5) dissolved in acetic acid (Sigma-Aldrich) was added to the mono-layer cultured in 6 well plates and then incubated for 30 min at RT. The excess of dye was removed by washing the wells three times with distilled water. 

Neutral mucins production was determined by Periodic Acid Schiff (PAS) staining. On the 21st day of culture, cells were fixed for 1 min with 0.5 mL/well of a formalin/ethanol fixative solution (1:10) and rinsed with water. Periodic acid (0.5 mL/well) was then added and kept for 5 min at RT. Dye in excess was removed and 0.5 mL of Schiff reagent were incubated for 15 min at RT. After washing with distilled water, the monolayers were observed under the optical microscope (Nikon TE 2000U, Amstelveen, The Netherlands), equipped with a digital camera DXM1200F (Nikon, Amstelveen, The Netherlands).

#### 2.6.2. Muc-5AC and Muc-2 Expression and Release

Mucin-5AC and mucin-2 expressions was analyzed by immunostaining with a confocal microscopy (Zeiss LSM 980, Oberrkochen, Germany). Cells were fixed with paraformaldehyde 3% for 30 min, rinsed with PBS, permeabilized with 0.1% Triton X-100 for 10 min, and blocked with 3% BSA for 60 min at RT. The cells were labeled with mouse monoclonal anti-Mucin 5AC antibody (1:100, Thermo-Fisher, Waltham, MA, USA) or mouse monoclonal anti-Muc-2 antibody (1:100, Thermo Fisher Waltham, MA, USA), incubated for 60 min at RT and labeled with Alexa Fluor 488 conjugate secondary antibodies. Nuclei were stained with DAPI. 

Mucin 5AC and Mucin 2 release in the medium were also quantified by Elisa (FnTest, Fine Biotech Co., Ltd. Wuhan, Hubei, China) according to the manufacturer’s instructions.

### 2.7. Electron Microscopy

Transmission Electron Microscopy (TEM) analysis was performed according to Bernardo et al (2021) [48] with slight modifications. Mono-cultures, bi-cultures and tri-cultures (established as described in paragraph 2.2) grown on inserts were fixed in 2.5% glutaraldehyde, 2% paraformaldehyde, 2 mM CaCl_2_ in 0.1 M sodium cacodylate buffer, pH 7.2, overnight at 4 °C. After washing, samples were post-fixed with 1% OsO_4_ in 0.1 M cacodylate buffer for 1 h at RT and treated with 1% tannic acid in the same buffer for 30 min. Post-fixed specimens were washed, dehydrated through a graded series of ethanol solutions (30–100% ethanol) and embedded in Agar 100 (Agar Scientific Ltd, Stansted Mountfitchet, UK). Insert membranes were detached from their supports by a razor and polymerized in a beam flat embedding mold for TEM. 

Ultrathin sections, obtained by an UC6 ultramicrotome (Leica), were stained with uranyl acetate and Reynolds’ lead citrate and examined at 100 kV by EM 208S TEM (FEI-Thermo Fisher Scientific, Eindhoven, The Netherlands), equipped with the Megaview II SIS camera (Olympus, Co., Shinjuku, Tokio, Japan).

For Scanning Electron Microscopy (SEM) analysis, cell cultures grown on inserts were fixed with 2.5% glutaraldehyde in 0.1 M sodium cacodylate buffer overnight at 4 °C. Samples were post-fixed with 1% OsO_4_ in 0.1 M sodium cacodylate buffer for 1 h at RT and dehydrated through a graded series of ethanol solutions (from 30% to 100%). Ethanol was gradually substituted by a 1:1 solution of hexa-methyl-disilazane (HMDS): absolute ethanol for 30 min, and successively by pure HMDS for 1 h (RT). Then, samples were completely dried removing the HMDS and left under the hood for 30 min. Dried samples were detached from the supports by a razor and mounted on SEM stubs, coated with gold (10 nm) and analyzed by a field emission GeminiSEM450 (ZEISS, Oberkochen, Germany) [49].

### 2.8. SiO_2_ NPs Dispersion Preparation and Characterization 

Ultra-stable red fluorescent SiO_2_ nanobeads (50 nm, Z-potential −45 ± 6 mV) (HiQ-Nano s.r.l. Arnesano, Lecce, Italy) were provided as stock solution in water at a concentration of 1mg/mL. As recommended by the provider, SiO_2_ NPs were sonicated for 5 min and vortexed for 1 min and resuspended in DMEM complete culture medium (*w*/*o* Phenol Red) with 1% heat-inactivated FBS at the final concentration of 100 µg/mL. The suspension was characterized by Dynamic Light Scattering (DLS) Zetasizer NanoS (Malvern Panalytical LtD, UK) determining Z average and PDI values. Measurements of NP suspension in H_2_O and in complete culture medium were performed at time 0 and after 24 h at 25 °C°. For each sample, 10 repeated measurements were collected.

### 2.9. SiO_2_ NPs Translocation and Uptake 

On day 21 of culture, the medium was removed and Ap and Bl chambers were washed twice with PBS. Fluorescent SiO_2_NPs (100 µg/mL) in 0.5 mL complete DMEM w/o phenol red containing 1% FBS were added to the Ap compartment, while 1 mL of fresh medium was added in the Bl compartment; inserts were incubated at 37 °C for 24 h. NP translocation was determined by spectrofluorometer (Nivo, Perkin Elmer, Waltham, MA, US) at λ 565 nm excitation and λ 590 nm emission by analyzing 100 μL of Bl medium, in triplicate for each insert. NPs uptake was analyzed by confocal microscopy on a Zeiss LSM 980. Cell monolayers were washed twice with PBS and fixed for 30 min with paraformaldehyde 3%. The samples were then rinsed with PBS, nuclei were counterstained with DAPI (Thermo Fisher Scientific, Waltham, MA, USA) for 10 min. Membranes were then removed with a scalpel and placed on glass slides, before mounting coverslips with Vectashield (Vector Laboratories) mounting medium.

### 2.10. Statistical Analysis 

Statistical analyses were performed with the SPSS software package (SPSS for Windows 14.0, SPSS, Chicago, IL, USA). One-way analysis of variance (ANOVA) was used to evaluate group comparison. If the group by each time interaction was significantly different (*p* < 0.05), differences between groups were compared with a post hoc test (Tukey’s). Data represents the average of three independent experiments.

## 3. Results

### 3.1. M Cell Phenotype Induction

#### 3.1.1. Raji B Exposure Conditions

M-cells are characterized by irregular brush border and a reduced glycocalyx; their origin as well as the regulation of their development is not yet fully defined.

Although many different protocols have been developed to obtain Caco-2 conversion into M cells, Caco-2/Raji B co-culture is the most controversial and critical aspect of the proposed model. A plethora of different conditions were used to promote this conversion ranging from: (i) direct contact between the two cell lines by adding in normal orientation Raji B cells to the basolateral compartment [21,27,38,41]; (ii) inverted models in which epithelial monolayers are grown in normal orientation for few days and then turned upside down; the Raji B cells are then added apically to an inverted insert sealed with silicone wrap in a petri dish [25,40]; (iii) use of conditioned medium [50]. None of these protocols are completely satisfactory so far. In the present study we attempted to optimize all these approaches, by identifying strengths and weakness of each of them, aiming to identify best and simplest experimental conditions. 

Moreover, although different attempts have been made to identify specific markers characterizing M-cells [21,22,41], to date no unequivocal indicator has been clearly identified. Frequently, this gap has been overcome by indirect evidence as, for instance, variation of barrier integrity or cytoskeleton and tight junction proteins perturbation. Furthermore, to determine how long the M-phenotype was maintained in our experimental condition after the last cycle of induction (19th day of culture), co-cultures were checked for barrier integrity until the next 5 days after the end of the induction cycle. We observed that the model was stable until the 22nd day of culture (data not shown).

#### 3.1.2. M-Cell Marker: Zonula Occludens (ZO-1) Expression 

**Figure 1:**
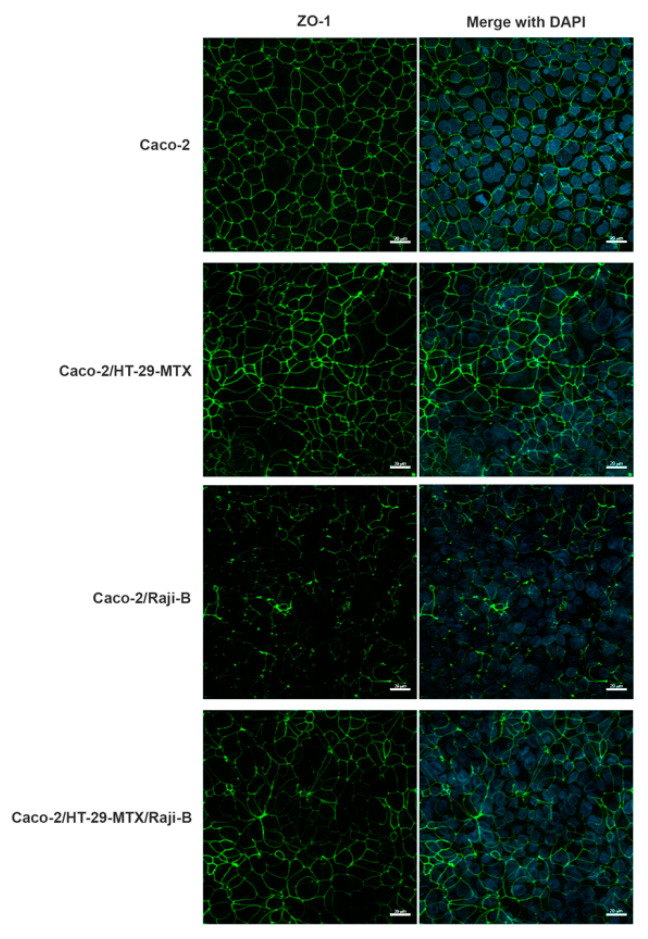
Analysis of ZO-1 expression (green) by confocal laser scanning microscopy (CLSM) in different (co)-culture conditions after 21 days of culture. Caco-2 mono-culture; Caco-2/HT29-MTX co-culture; Caco-2/Raji B co-culture; Caco-2/HT29-MTX co-culture; Caco-2/HT29-MTX/Raji B tri-culture. Nuclei were counterstained with DAPI (blue). Bars, 20 µm.

To investigate M-phenotype induction, the labelling of *ZO-1*, a marker of TJ integrity, was performed in the different (co)-culture conditions. As reported in Figure 1, an evident perturbation of this protein network is observed in all the co-culture conditions providing indirect evidence of the presence of M-cells. For Caco-2/HT29-MTX co-culture, the masking of the fluorescent signal can be attributed to the presence of the mucus layer.

#### 3.1.3. M Cell Marker: Wheat Germ Agglutinin (WGA)

**Figure 2:**
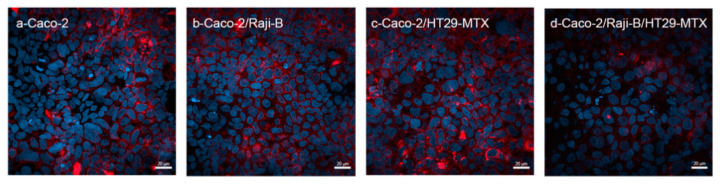
CLSM analysis of WGA expression (red) in the different (co)culture conditions at 21st day of culture. Caco-2 mono-culture; Caco-2/Raji B co-culture; Caco-2/HT29-MTX co-culture; Caco-2/HT29-MTX/Raji B tri-culture. Nuclei were counterstained with DAPI (blue). Bars, 20 μm.

As reported in Figure 2, WGA staining fails to represent a specific marker for M-cells, since it is observed in all culture conditions. It is noteworthy that, in the tri-culture Caco-2/HT29-MTX/ Raji B and in the bi-culture Caco-2 /Raji B conditions the signal is less intense, confirming that M-cells display a reduced glycocalyx, according to the evidence that WGA has a high binding affinity to sialic acid and N-acetylglucosamine sugar residues [51].

#### 3.1.4. Ultrastructural Characterization

**Figure 3:**
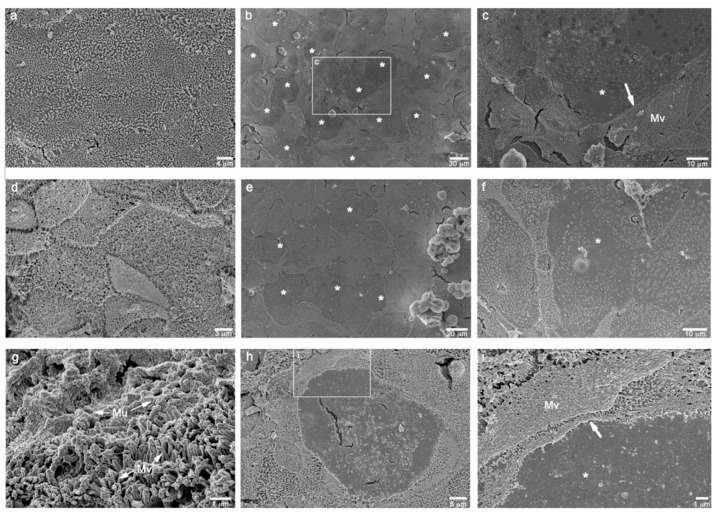
SEM micrographs of the different combinations of cell cultures grown on inserts, at 21st day of culture. (**a**): Caco-2 monoculture showing a homogeneous leaflet of polarized epithelial cells with a great abundance of surface microvilli. (**b**): Caco-2/Raji B co-culture displaying some rounded areas of microvilli loss. (**c**): high magnification of the squared area in b showing the boundary (white arrow) between presence (Mv) and absence (*) of the surface microvilli. (**d**): Caco-2/HT29-MTX differentiated co-culture rich in surface microvilli. (**e**,**f**): Caco-2/HT29-MTX/Raji B tri-culture showing a lot of inducted smooth rounded areas of possible M-cell phenotype (*). (**g**): Caco-2/HT29-MTX co-culture displaying a consistent mucus layer (Mu) on the microvilli. (**h**,**i**): high magnifications of a feasible M-cell phenotype (*) in Caco-2/HT29-MTX/Raji B tri-culture (white arrow: boundary of the microvilli loss.

Ultrastructural analysis of the differentiated tri-cultures by scanning electron microscopy (SEM) confirmed the growth of a polarized epithelial leaflet characterized by a rich brush border, typical of the intestinal barriers, and the formation of an apical region versus a basolateral one. As previously reported, no specific markers for human M-cells have been clearly identified; however, tri-cultures electron microscopy analysis undoubtedly confirmed the ability of Raji B cells, or their conditioned medium, to induce numerous regions of totally loss of surface microvilli (considered a distinctive sign of an M-cell phenotype) (Figure 3). 

In particular, the mono- and co-cultures of Caco-2 and Caco-2/MTX cells, respectively, were both well-differentiated polarized cell leaflets, rich in surface microvilli and the latter one showing extended zones of mucus (Figure 3a,d,g). The addition of Raji B cells to the basolateral side of both the models clearly showed the induction of an M-cell phenotype in several, and not consecutive, round surface areas, suggesting that there was transformation of a subpopulation of epithelial cells (Figure 3b,c,e,f,h,i). 

**Figure 4:**
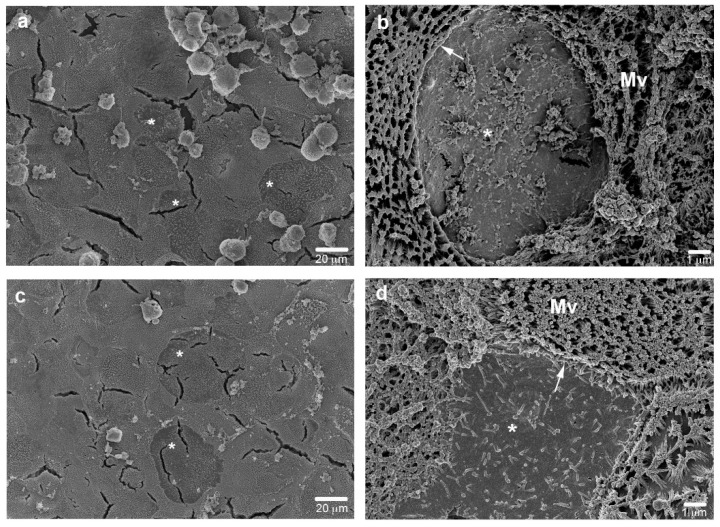
Caco-2 (**a**,**b**) or Caco-2/HT29-MTX cultures (**c**,**d**) grown in Raji B conditioned medium, at 21st day of culture. SEM micrographs show the ability of the only conditioned medium to induce the loss of surface microvilli both in Caco-2 (**b**) or Caco-2/HT29-MTX cultures (**d**). Bars a and c 20 μm; b and d 1 μm.

Results from the addition of Raji B conditioned medium to the basolateral side of the co-cultures showed the same M-cell phenotype induction in the epithelial leaflet surface, although in a lesser extent (Figure 4), suggesting that soluble mediators are involved in the M-cell conversion, although their action on the morphological changes is less effective.

**Figure 5:**
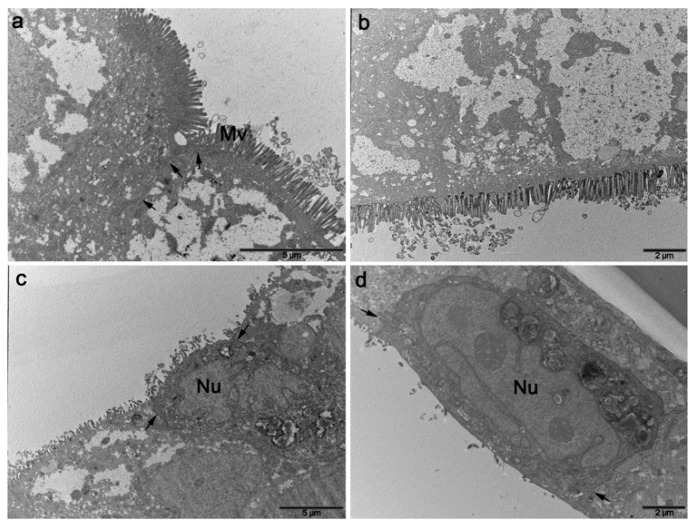
TEM micrographs of: (**a**) Caco-2 monoculture, (**b**) Caco-2/HT-29 MTX co-culture, (**c**) Caco-2/Raji B co-culture, and (**d**) Caco-2/HT-29 MTX/Raji tri-culture at the 21st day of culture. Differentiated epithelial cells show the typical brush border and TJ of the intestinal barrier (**a**,**b**) while, in the tri-culture with Raji B cells, cells are characterized by the loss of microvilli (mV) displayed always a more electrodense cytoplasm and the absence of the typical electron transparent accumulation of amorphous material of Caco-2 cells. Bars a and c 5 μm; b and d 2 μm.

Transmission electron microscopy analysis of transversal ultrathin sections of the co-cultures onto inserts highlighted a strong polarization of the differentiated intestinal cells revealing the set-up of well-organized TJ and microvilli (Figure 5). The M-cell phenotype in the Raji B induced leaflets was rarely observed, as the method was not adequate to the frequency of the event, but when cells without microvilli were found, they were characterized by a reduced and more electrodense cytoplasm (Figure 5c,d). These cells appeared morphologically different but it was not possible to find other specific ultrastructural features. No differences were observed in the monolayers grown on 1 or 3 µm pore size inserts (reported in Supplementary Figure S1). 

### 3.2. Barrier Integrity 

#### 3.2.1. Transepithelial Electrical Resistance (TEER)

Integrity of the mono and (co)-culture models was evaluated by TEER measurements at the 20th and the 21st days of culture. Before TEER determination, inserts were relocated in a new multi-well plates, to reduce any confounding factors due to presence of Raji B cells or cellular debris in Bl compartment. TEER values for Caco-2 monoculture and Caco-2 /HT29MTX (9:1 ratio) grown on *n* 1 µm and 3 µm pore size inserts were monitored up to 21 days to check the differentiation process (Supplementary Figure S2). 

**Figure 6:**
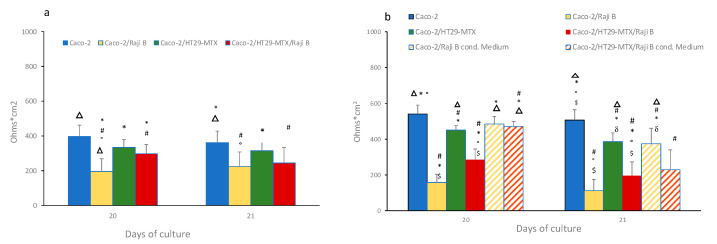
TEER evaluation in 1 (**a**) and 3 (**b**) µm pore size insert at 20th and 21st days of culture. Results are the mean of three separate experiments performed in triplicate. Statistical analysis has performed by One-way Anova Test followed by post hoc test (Tukey’s (*p* < 0.05) to evaluate group comparison. # versus Caco-2, * versus Caco-2/RajB; ° versus Caco-2/HT29-MTX; Δ versus Caco-2/HT29-MTX/ Raji B, $ versus Caco-2/Raji B medium; δ versus Caco-2/HT29-MTX/ Raji B medium.

As shown in Figure 6a, on 1 µm pore size inserts a drastic TEER reduction was observed in the Caco-Raji B co-culture in respect to the Caco-2 monoculture and Caco-2/HT29-MTX co-culture conditions (195 ± 47 against 396 ± 63 and 334 ± 44 ohms × cm^2,^ respectively) at the 20th day of culture immediately after the end of lymphocyte stimulation. A slight recovery was observed at the 21st day even if not significant. The tri-culture condition displayed a milder decreased TEER value (297 ± 53 ohms × cm^2^) compared to the Caco-2/ Raji B system, still significantly lower than Caco-2 monocultures.

When the cells are cultured on 3 μm pore size inserts higher difference between the Caco-2 monoculture (540 ± 50 ohms × cm^2^) and the Raji B co-cultures were reported; in fact, in the Caco-Raji B condition, a dramatic drop of TEER values was registered (168 ± 24 ohms × cm^2^), while in the tri-culture TEER values (285 ± 60 ohms × cm^2^) came closer to in vivo values of human intestinal barrier.

The effect of Raji B conditioned medium on barrier integrity has also been investigated. As shown in Figure 6b, conditioned medium caused a very slight decrease TEER both in the Caco-2 cells (485 ± 42 ohms × cm^2^) and in Caco-2/HT29-MTX co-culture (470 ± 30 ohms × cm^2^) that is more pronounced at the 21st day. 

#### 3.2.2. FITC-Dextran and Lucifer Yellow Passage 

**Figure 7:**
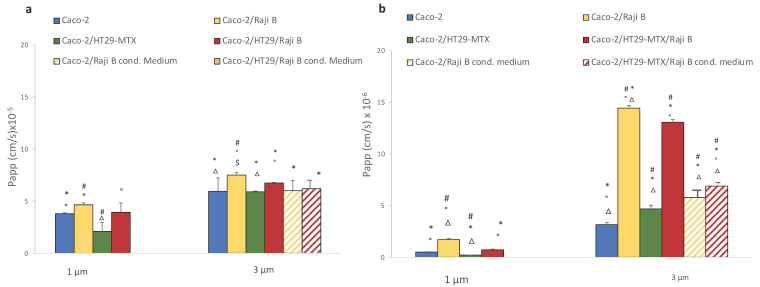
FITC-dextran (**a**) and LY translocation (**b**) in 1 and 3 µm pore size inserts at 21st day of culture. Results are the medium of three separate experiments performed in triplicate. Statistical analysis was performed by One-way Anova Test followed by post hoc test (Tukey’s (*p* < 0.05) to evaluate group comparison. # versus Caco-2, * versus Caco-2/RajB; ° versus Caco-2/HT29-MTX; Δ versus Caco-2/HT29-MTX/ Raji B, $ versus Caco-2/Raji B medium; δ versus Caco-2/HT29-MTX/ Raji B medium.

Barrier permeability was assessed by passive passage of paracellular markers FITC-dextran 40 KD and LY (457 D) at the 21st day of culture on 1- and 3- µm pore size inserts. An increase of passage of both markers is reported in presence of Raji B cells (Figure 7a,b) particularly noticeable on 3 µm pore inserts, confirming that they are a more suitable support for M-phenotype induction. 

In particular, FITC dextran passage has a Papp value of 3.79 × 10^−5^ ± 0.01 cm/sec for Caco-2 monolayer on 1 µm pore size insert instead of 5.95 × 10^−5^ ± 0.2 cm/sec when cells are cultivated on 3 µm pore size insert. Caco-2/HT-29 MTX condition with respect to the Caco-2 monoculture showed similar Papp values, (2.1 × 10^−5^ ± 0.8 cm/sec for 1 mm and 5.9 × 10^−5^ ± 0.06 cm/sec for 3 mm). Conversely, an increase passage in the Caco-2/Raji B condition was observed (4.65 × 10^−5^ ± 1.2 cm/sec for 1 µm and 7.6 × 10^−5^ ± 0.2 cm/sec for 3 µm). In the triple-culture condition, there was a milder passage in respect to the Caco-2/Raji B bi-culture, confirming that the presence of mucus is able to ameliorate the model by mitigating the barrier permeability.

The same scenario has been observed for LY passage. Even in this case, the highest translocation rate is obtained with the coculture Caco-2/Raji B condition compared to the Caco-2 monoculture (1.7 × 10^−6^ ± 0. 1 cm/sec versus 0.5 × 10^−6^ ± 0.03 cm/sec for 1 µm and 14.4 × 10^−6^ ± 0.03 cm/sec versus 3.15 × 10^−6^ ± 0.2 cm/sec for 3 µm pore size inserts). Again, in the triple co-culture the strong impairment of the barrier caused by lymphocytes is mitigated (0.7 × 10^−6^ ± 0.06 cm/sec for 1 µm and 13 × 10^−6^ ± 0.3 cm/sec for 3 µm pore size inserts). As observed for TEER values, Raji B conditioned medium is less effective in both cases for FITC dextran and LY in increasing barrier permeability, which is an indirect proof of its lesser capacity in inducing the M-phenotype in Caco-2 cells. Moreover, in the present experimental conditions, LY has been shown to be a more sensitive and reproducible marker

### 3.3. Mucus Characterization 

#### 3.3.1. Mucus Staining 

Caco-2 cells /HT29-MTX co-cultured at a 9:1 ratio provide a model where absorptive and globet cells are present simultaneously, comprising small clusters of HT29-MTX embedded in Caco-2 absorptive cells [52]. The co-culture consists of polarized monolayers that are morphologically and functionally similar to the native epithelium. According to literature evidence, a 9:1 ratio (Caco-2:HT29-MTX respectively) was revealed as the optimal co-culture ratio for permeability studies application [53,54]. This cell seeding ratio reproduces the in vivo balance between epithelial cells and globet cells allowing maintenance of good functionality of the in vitro barrier (e.g., TEER values), although it is reported that the mucus secreted in these in vitro cultures is thinner compared to the mucus layer found on human explants (approximately 5 mm versus 600 mm thick [55].

**Figure 8:**
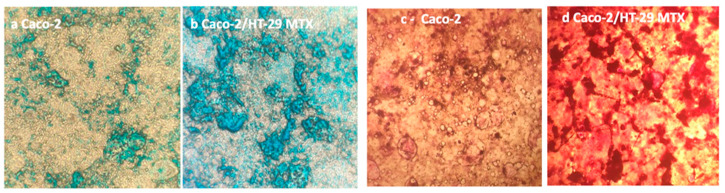
Alcian blue staining in: (**a**): Caco-2 monoculture and (**b**): Caco-2/HT29-MTX co-culture. Periodic Acid Schiff staining in: (**c**): Caco-2 monoculture and (**d**): Caco-2/HT29-MTX at 21st day of culture on 6 multi-well plates. Magnification 200×.

To assess mucus production, Caco-2 /HT29-MTX co-cultures were seeded at 9:1 ratio on 6-well plates and left to differentiate for 21 days. At the end of this period, both cultures were stained with Alcian blue for acidic mucins and Periodic Shiff for neutral ones. As shown in Figure 8, both staining methods confirmed the production of mucus byHT29-MTX cells by forming a layer on top of the epithelial cells.

#### 3.3.2. MuC-2 and MuC5 AC Expression and Release

**Figure 9:**
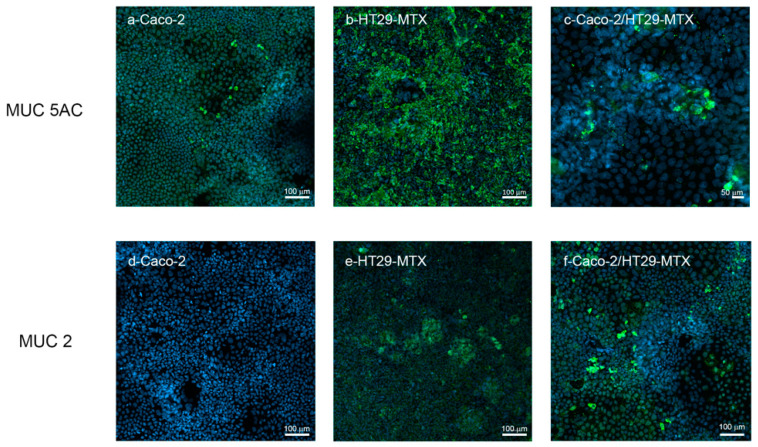
MUC 2 and MUC 5 AC (green) labelling in Caco-2 monoculture, HT29-MTX monoculture and Caco-2/HT29-MTX co-culture analyzed by confocal microscopy. The cells were counterstained with Hoechst 33258 (blue) for nuclei identification.

HT29-MTX represent a homogeneous subpopulation selected by adapting to 10–5 methotrexate and are able to produce different mucins such as MUC-2, MUC-AC and MUC5B [35]. As reported in Figure 9, Caco-2, HT29-MTX and co-culture Caco-2/HT29-MTX at a 9:1 ratio were compared for MUC-2 and MUC-5AC expression by confocal microscopy. HT29-MTX shows an intense signal for MUC-5 AC compared to Caco-2 monoculture. A similar signal is observed for MUC-2, which is rarely expressed by Caco-2 cells and more consistently by HT29-MTX cells.

The bi-culture provides some clusters of expression both for MUC-2 and MUC-5AC, confirming the formation of a mucus layer in our experimental culture condition. 

**Figure 10:**
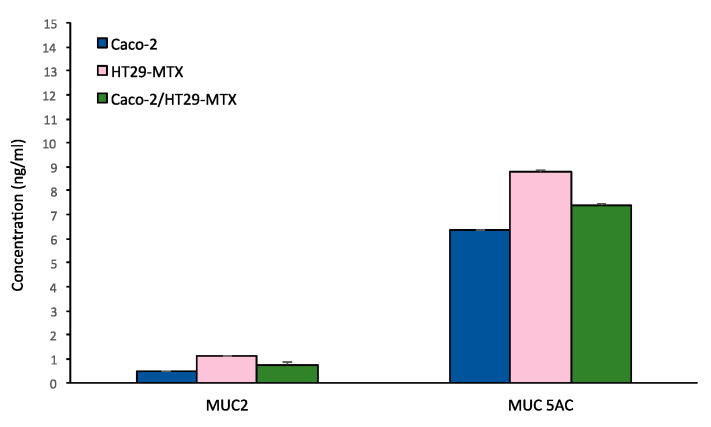
MUC 2 and MUC 5 AC mucins release in Caco-2 monoculture, HT29-MTX monoculture and Caco-2/HT29-MTX co-culture.

Characterization of the secreted gel forming mucins MUC-2 and MUC-5AC release in the medium was investigated by ELISA assay both in the monocultures and in the bi-culture Caco-2 /HT29-MTX, as shown in Figure 10.

MUC5 AC release was abundant compared to MUC-2 in all the culture conditions, with a higher production for HT29-MTX monoculture. 

### 3.4. SiO_2_ NPs Uptake and Translocation as a Case Study for Tri-Culture Model Efficiency 

#### 3.4.1. SIO_2_ NPs Characterization by DLS 

Fluorescent SiO_2_ nanobeads were characterized by Dynamic Light Scattering (DLS), which provides the hydrodynamic diameter and the agglomeration state of the samples, referred to as Z-average (Z-ave) and polydispersity index (PDI), respectively. The SiO_2_ nanobeads, diluted in DMEM without Phenol Red supplemented with 1% FBS, were analyzed at a concentration of 100 µg/mL, before performing each experiment (time 0) and after 24 h. In Table 1, Z-ave and PDI values of SiO_2_ dispersions in water and DMEM at time 0 and in DMEM after 24 h of exposure are reported.

Increase of the Z-ave in respect to the nominal NPs dimension (50 nm) is observed in water dispersion; it can be attributed to an increase in the hydrodynamic radius due to the behaviour of the charged particles in a polar aqueous environment rather than to a NPs agglomeration. The dispersion in the treatment medium (DMEM plus 1% FCS) generates limited particle aggregation that remains constant during the following 24 h. PDI value also increase in DMEM dispersion in respect to water dispersion but, anyway, only single particle size population has been identified. After 24 h, particle size distribution is quite worse and a small peak is detected on the left of the main peak, probably caused by experimental artefacts or small protein aggregates. Graphs of population size distribution for intensity have been added as supplementary material (Figure S3).

Moreover, further experiments with stable particles are needed to assess the validity of the proposed model.

#### 3.4.2. Uptake and Translocation 

**Figure 11:**
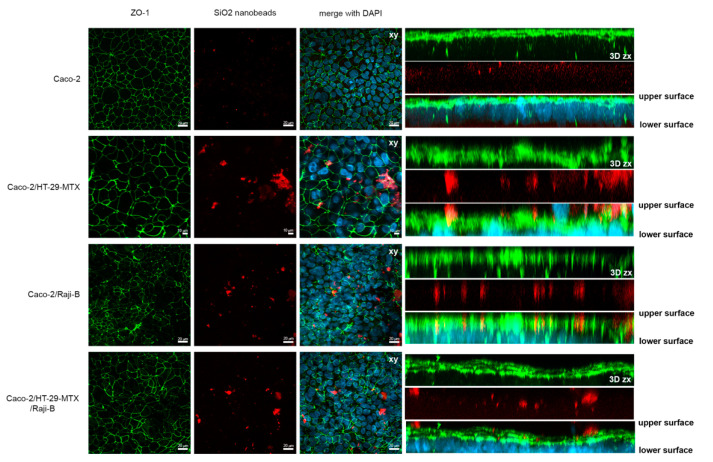
SiO_2_ nanobeads (red) internalization in Caco-2 monoculture, Caco-2/HT29-MTX co-culture, Caco-2/Raji B co-culture, and Caco-2/HT29-MTX/Raji B tri-culture on 3 µm pore insert. Cells were counterstained with DAPI (blue) and ZO-1 (green). Representative 3D reconstructions (Z-stacks) by CLSM are reported. 50–70 Z-slices were acquired every 0.20 µm covering the total height of the cell cultures on inserts. Left panels show xy Z-stack projections, while right panels show xz Z-stack projections; the upper and the lower surface of inserts are indicated. Scale bars are 20 and 10 µm, as indicated.

To highlight the different behaviors of the (co)culture models toward nanoforms, treatments with SiO_2_ fluorescent NPs were performed at 100 µg/mL for 24 h evaluating internalization by confocal microscopy and translocation by fluorometric analysis. As shown in Figure 11, nano silica NPs are visible on the extra cellular surface of Caco-2/HT29-MTX co-culture, probably entrapped by mucus. Some particles were also noted on the Caco-2/Raji B cells co-culture, where mucus is absent but microvilli-free regions are present in correspondence with M-cells. 

Furthermore, a ZO-1 perturbation *n* in all the culture conditions following 24 h exposure to 100 μg SIO_2_ is observed.

**Figure 12:**
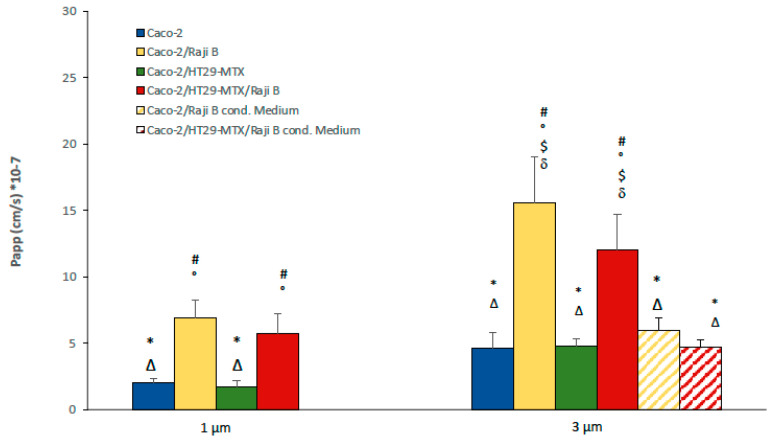
SiO_2_ NPs translocation in Caco-2 monoculture in the different (co)-culture conditions on 1 µm and 3 µm pore size inserts. On 3 µm pore, insert data on silica translocation in presence of Raji B conditioned medium are also reported. # versus Caco-2, * versus Caco-2/RajB; ° versus Caco-2/HT29-MTX; Δ versus Caco-2/HT29-MTX/ Raji B, $ versus Caco-2/Raji B medium; δ versus Caco-2/HT29-MTX/ Raji B medium.

As reported in Figure 12, the highest translocation rate was determined in the Caco-2/Raji-B culture followed by the tri-culture model, suggesting that M-cells act as portals for NPs cell crossing. 

The impact of pore inserts dimension is evident even in this case, with a magnification of the effect on 3 µm pore inserts. Passage through empty filters has been analyzed (Supplementary Figure S4) to evaluate the impact of the different pore size.

Conditioned medium from Raji B cells in co-culture with both Caco-2 and Caco-2/HT29-MTX cells proved to be less efficient in allowing the nano silica translocation, confirming the limited performances of this model.

## 4. Discussion

The first in vitro model of human FAE was developed by Kerneis and co-authors (1997) [56] by co-culturing Caco-2 cells with isolated lymphocytes from mouse Peyer’s patches. Previous in vivo murine models showed that the intravenous injections of Peyer’s patches lymphocytes in immunodeficient mice resulted in new formation of lymphoid follicles and follicle-associated epithelium with typical M-cells [57]. Several years later, Gullberg et al (2000) [27] proposed a model culturing Caco-2 cells with human B lymphocytes, while in another study [58] inserts were inverted to promote accessibility and contact among lymphocytes and intestinal epithelial cells. The same model, although very laborious, was applied by des Rieux et al [40] in pilot studies with NPs used to assess the functionality of the in vitro barrier. Study results indicate that NPs were preferentially transported through the M-cell. Recently the application opportunities of advanced in vitro models of intestinal barrier with respect to Caco-2 monolayer for NPs uptake and translocation raised much interest in many research groups. 

Intestinal co-culture with mucus secreting cells and M-cells induction by lymphocytes resembles the in vivo intestinal mucosa, since it includes crucial elements for NP internalization such as the presence of mucus, which actively interacts with NPs entrapping or facilitating their passage, and of M-cells that have a primary role in the sampling and traffic of particulate matter. 

As reported above, available studies from the literature are patchy and diluted over time. So further efforts are needed to harmonize the various protocols developed, aiming to find the most suitable experimental conditions for improving the protocol transferability between laboratories.

In this respect, many efforts have been dedicated to defining the conditions of stimulation of M-cells as well as to the identification of specific markers of this conversion. In our opinion, this remains the aspect of the model that needs to be optimized and standardized even more. For instance, it must be definitely clarified whether the different cycles of stimulation by Raji B cells need the addition of fresh cells every time, or whether they can remain for the whole stimulation period. According to our results the best condition to obtain M cell phenotype induction is by co-culturing Caco-2/HT29 –MTX at 9:1 ratio from the 14th day of culture and adding Raji B cells for 5 days in the Bl compartment, refreshing them three times and allowing the inserts to rest 24 h before any treatments and TEER measurement. We observed that the tri-culture model was stable for 48 h from the end of lymphocyte induction, so it is recommended to perform the experiments no later than this period.

Moreover, we observed that presence of Raji B cells in the Bl compartment interferes with the barrier integrity measurements (particularly for TEER determination), so we advise the removal of lymphocytes from the Bl compartment before any evaluation of barrier integrity, moving the inserts in a clean multi-well plate. 

Results obtained with Raji B conditioned medium, in terms of functionality of the barrier (Figure 6B) SEM analysis (Figure 4) and nanoparticle translocation (Figure 12), were less efficient compared to the traditional model and this protocol is not suggested for further applications. 

Many efforts have been made to determine the reliable cellular markers of human M-cells, but to date this marker has not yet been identified. In vivo models’ identification of M-cells has been more clearly proven. For instance, in murine models, intestinal M-cells were stained by *Ulex euroaeus* agglutinin (UEA, 1) but were negative for WGA (Wheat germ agglutinin) [59]. Conversely, in in vitro studies this identification is much more ambiguous. WGA was used for M-cells identification [22] while in other papers it was used for mucus staining [41].

Our results indicate that WGA staining fails to be a specific and simple marker to identify enterocytes conversion to M-cells. In our opinion, ultrastructural investigation by TEM and SEM is to date the best approach to verify M-cells presence.

In the present study, an extensive characterization of the barrier integrity of the (co)-culture model has been performed. We confirm what is reported by several authors [41], i.e., the presence of HT29 and Raji B cells increases the barrier permeability, allowing better reproduction of the epithelium of the human gut. Both TEER and LY have proven to be simple and reproducible indicators of barrier performances, also useful to quickly check the acceptability of the model before NPs treatment. In our experimental conditions, a TEER value below 150 ohms × cm^2^ and a LY Papp value > 15 × 10^−6^ are considered not acceptable. 

Regarding the impact of the trans-well insert pore dimension on barrier function, our data indicate that, for NPs translocation studies, 3 µm pore dimension is preferable since it ensures a greater possibility of particle passage even in conditions of NP aggregation. TEM images confirm that there is no passage of epithelial cells thorough the filter membrane (Supplementary Figure S1). 

Production of a mucus layer in 9:1 co-culture of Caco-2/HT29-MTX has been confirmed by Alcian blue and Periodic Shiff staining. Both methods are fast and simple so they can be proposed as routine methods to verify the presence of the mucus layer in the (co)-culture. The more refined characterization of mucins presents in the mucus layer by confocal microscopy and by secreted gel forming mucins, clarifying that both MUC-2 and MUC-5AC are expressed, the latter in larger quantity. Mucus characterization is relevant in predicting the possible interactions with NPs which may affect their barrier penetration capacity. In the near future the possibility of using synthetic mucus with controlled composition could provide an additional useful element for model standardization [60].

The parameters and indicators considered in the study are summarized in Table 2.

The SiO_2_ case study has provided interesting confirmation on the suitability of the proposed approach. An evident effect of the mucus layer in entrapping the NPs is highlighted in Figure 11, where the red NP spots are clearly observable in the presence of HT29-MTX (Figure 11). This result agrees with what was reported by Garcia Rodriguez and co-authors [41] on a similar tri-culture model. They observed by confocal analysis that a considerable number of NPs remained in the apical side of the membrane, detained between microvilli and the mucus matrix. Moreover, a slight perturbation of ZO-1 is observed in all the culture conditions, indicating an alteration of the integrity of the epithelial barrier. Further experiments are needed to clarify this point. Despite the sieve action of the mucus, SiO_2_ NPs are also translocated through the cells, although in small quantity, in all the culture conditions, according to the following order: Caco-2/Raji B > Caco-2/HT29-MTX/Raji B > Caco-2/HT29-MTX Caco-2 (Figure 12). These data provide evidence of the fundamental role played by M-cells in the internalization of these NPs. Moreover, 3 µm pore inserts enhance the NP translocation and highlight the differences between the different culture conditions. Finally, once again results obtained with conditioned medium from Raji B cells are lower than expected. 

## 5. Conclusions

In conclusion, the tri-culture model Caco-2/HT29-MTX/Raji B is confirmed to be a reliable model for investigation of NPs interaction with intestinal epithelium. In this study some of the critical points related to its developing and functionality have been addressed to improve its standardization. In summary, the main points raised are:

- M cell phenotype induction: the best protocol is to co-culture Caco-2/HT29 –MTX at 9:1 ratio and, from the 14th day of culture, to add Raji B cells for the following 5 days in the Bl compartment in RPMI/DMEM (1:1) medium, refreshing them for three times. Inserts are put to rest for the next 24 h before any experimental activity. Model is stable for about 48 h;
- M cell marker: no specific marker for M cells has been identified. Electron microscopy (SEM or TEM) is now the unique way to identify them but further investigations are required to find a more practical and cheaper approach;
- Determination of barrier integrity is an important parameter of the model, easy and fast to measure. Both TEER and LY are simple and reproducible indicators of this parameter and, based on present data and literature evidence, the following acceptance criteria are proposed: TEER values must not be below 150 ohms × cm^2^ and the LY Papp value must not be greater than 15 × 10^−6^.
- Insert pore size is important aspect for NPs translocation experiments; present data indicate that 3 µm pore dimension is preferable;
- Co-culture of Caco-2/HT29 –MTX at 9:1 ratio produces an abundant mucus layer in which MUC-5AC are predominant. Alcian blue and Periodic Shiff staining can be used as routine method to verify the presence of the mucus layer in the (co)-culture.

The present study intends to provide a useful and standardized tool for researchers and for transferability of the model for regulatory purposes. It certainly can be improved and implemented with other cellular types such as, for instance, immunocompetent cells to bring us closer to the complexity of the intestinal mucosa. 

## Figures and Tables

**Table 1:** DLS characterization. Z average and PDI values (±SD) of fluorescent SiO_2_ nanobeads dispersion (100 µg/mL).

Dispersant	Time (Hours)	Z-Ave ± SD (nm)	PDI ± SD
H_2_O	0	82. ± 2	0.09 ± 0.01
DMEM 1%FCS	0	155 ± 2	0.22 ± 0.02
	24	125.7 ± 2	0.29 ± 0.08

**Table 2:** Summary of the main parameters and indicators investigated in the study.

Parameters	Indicators	Protocol Optimization
Transwell support	Insert pore size	3 µm pore inserts more suitable for NPs translocation studies
Barrier integrity	TEER and LY	Yes, acceptance criteria proposed
Mucus production	Alcian blue and Periodic Schiff staining	Yes, but only qualitative data
M-cell phenotype induction	Biochemical markers, TEM/SEM images	Yes, for TEM/SEM investigation (qualitative data) No specific biochemical markers identified

## Data Availability

Not applicable.

## Supplementary Materials

The following supporting information can be downloaded at: https://www.mdpi.com/article/10.3390/cells11213357/s1, Figure S1: TEM sagittal image of Caco-2 monoculture cultured on 3 μm filter; Figure S2: TEER measurement at different days of culture in 1 and 3 μm pore size inserts both for Caco-2 monolayer and Caco-2/HT-29 MTX co-culture; Figure S3: SiO_2_ nano beads graphs of population size distributionfor intensity obtained by DLS analysis; Figure S4: SiO_2_ Translocation studies through empty filters and cell monolayers.
