# Supplementary material for: Towards the Standardization of Intestinal In Vitro Advanced Barrier Model for Nanoparticles Uptake and Crossing: The SiO2 Case Study
Source: Cells. 2022 Oct 25;11(21):3357. doi: 10.3390/cells11213357 (PMC9654320; doi:10.3390/cells11213357)
Supplement: Supplementary file 1 [file cells-11-03357-s001.zip › cells-1919594-supplementary.pdf]

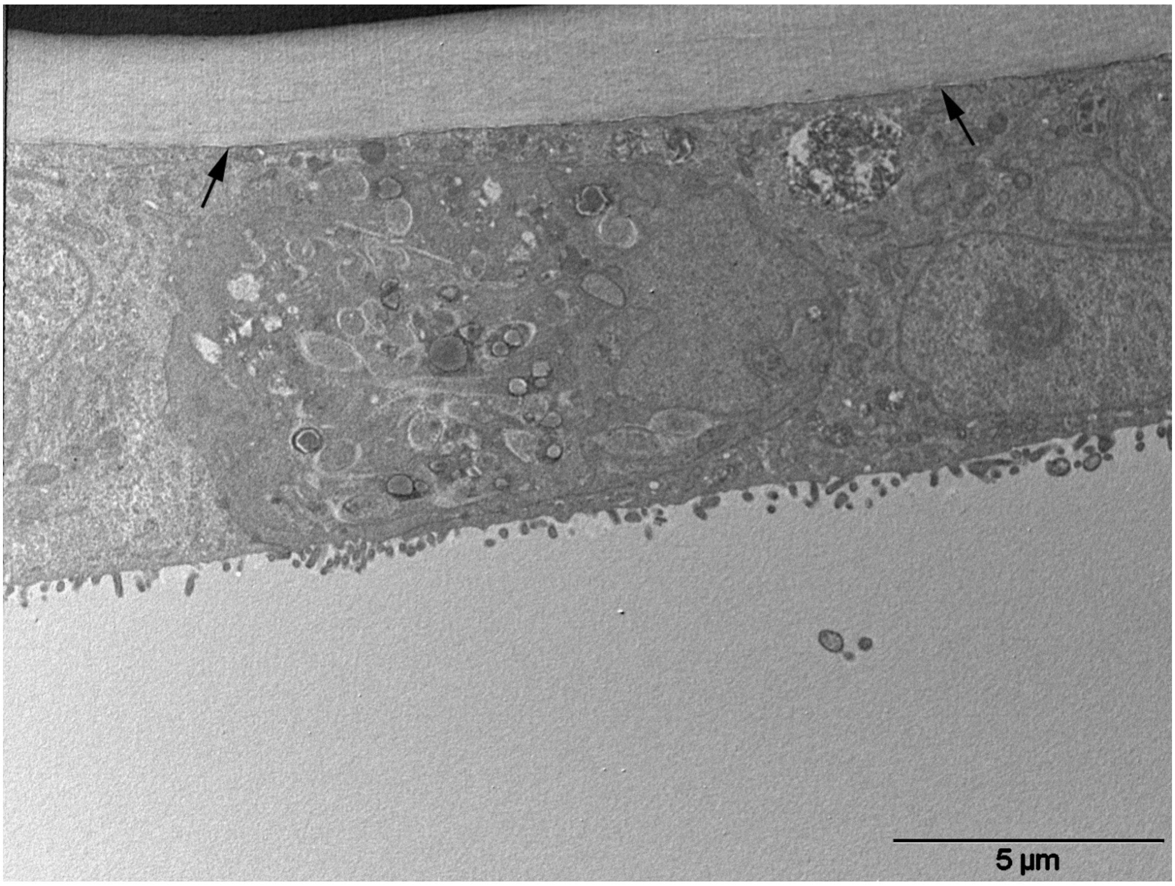

Figure S1. TEM sagittal image of Caco-2 monoculture cultured on 3 μm filters.

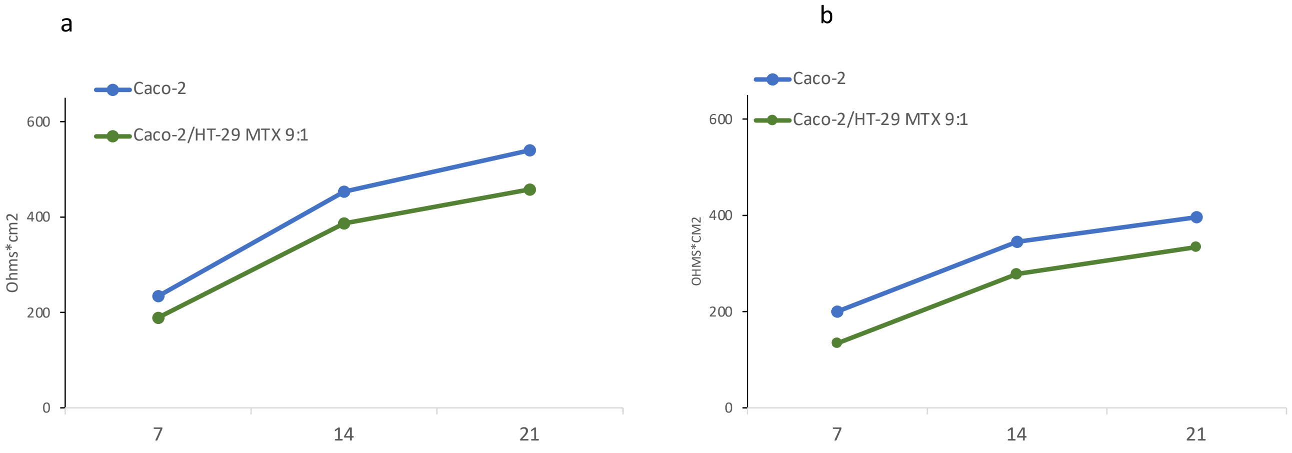

Figure S2. TEER measurement at different days of culture in 1 and 3 μm both for Caco-2 monolayer and Caco-2/HT-29 MTX co-culture.

H20 To

			Size (d.nm):	% Intensity:	St Dev (d.nm):
Z-Average (d.nm):	86,97	Peak 1:	94,65	100,0	27,62
PdI:	0,089	Peak 2:	0,000	0,0	0,000
Intercept:	0,945	Peak 3:	0,000	0,0	0,000
Result quality :	Good				

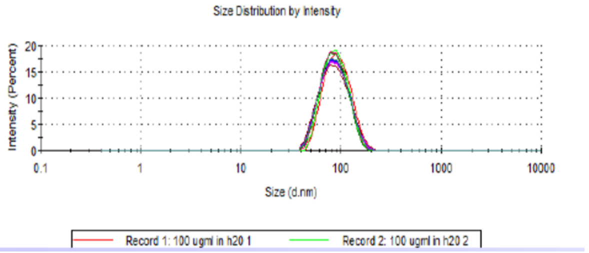

DMEM To

			Size (d.nm):	% Intensity:	St Dev (d.nm):
Z-Average (d.nm):	160,3	Peak 1:	206,6	100,0	106,6
PdI:	0,234	Peak 2:	0,000	0,0	0,000
Intercept:	0,966	Peak 3:	0,000	0,0	0,000
Result quality :	Good				

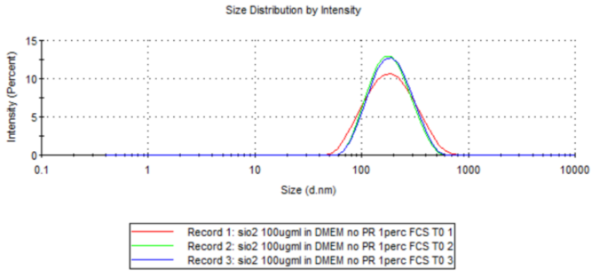

DMEM T24

			Size (d.nm):	% Intensity:	St Dev (d.nm):
Z-Average (d.nm):	126,2	Peak 1:	178,0	100,0	69,96
PdI:	0,291	Peak 2:	0,000	0,0	0,000
Intercept:	0,948	Peak 3:	0,000	0,0	0,000
Result quality :	Refer to quality report				

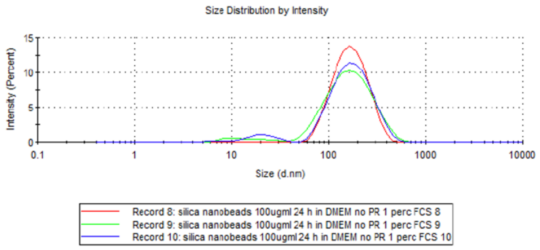

Figure S3. SiO_2 nano beads graphs of population size distribution for intensity obtained by DLS analysis:

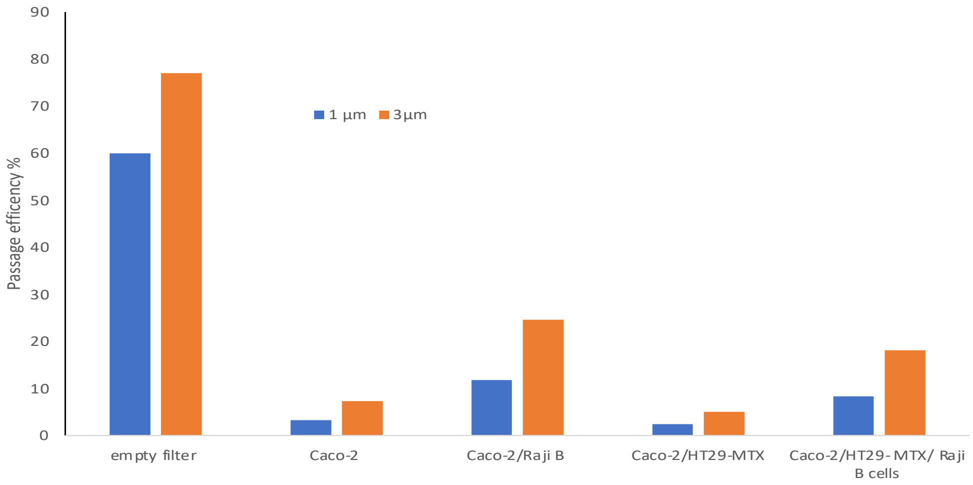

Figure S4. SiO_2 Translocation studies title through empty filters and cell monolayers
